# Patient activation in older people with long-term conditions and multimorbidity: correlates and change in a cohort study in the United Kingdom

**DOI:** 10.1186/s12913-016-1843-2

**Published:** 2016-10-18

**Authors:** Amy Blakemore, Mark Hann, Kelly Howells, Maria Panagioti, Mark Sidaway, David Reeves, Peter Bower

**Affiliations:** 1 NIHR School for Primary Care Research, Manchester Academic Health Science Centre, University of Manchester, Oxford Road, Manchester, M13 9PL UK; 2 Salford Royal NHS Foundation Trust, Salford Royal Foundation Trust, Stott Lane, Salford, M6 8HD UK; 3 NIHR Greater Manchester Primary Care Patient Safety Translational Research Centre, Manchester Academic Health Science Centre, University of Manchester, Oxford Road, Manchester, M13 9PL UK

**Keywords:** Patient activation, Depression, Long-term conditions, Multimorbidity, Self-management, Health-related quality of life

## Abstract

**Background:**

Patient Activation is defined as the knowledge, skill, and confidence a patient has in managing their health. Higher levels of patient activation are associated with better self-management, better health outcomes, and lower healthcare costs. Understanding the drivers of patient activation can allow better tailoring of patient support and interventions. There are few data on patient activation in UK patients with long-term conditions.

**Methods:**

A prospective cohort design was used. Questionnaires were mailed to 12,989 patients over the age of 65 years with at least one long-term condition in Salford, UK. They completed the Patient Activation Measure and self-report measures of: depression, health literacy, social support, health-related quality of life, and impact of multimorbidity. We report descriptive data on baseline activation and change over time, and use multivariate regression to model associations with patient activation at baseline and predictors of change in Activation over 6 months.

**Results:**

The cohort included 4377 (33.6 %) older people, of whom 4225 were mailed a further questionnaire at 6 months; 3390 returned it complete (80.2 %). At baseline, 15 % self-reported PAM level 1, 16 % level 2, 45 % level 3, and 25 % level 4. Across all patients, depression had the strongest association with patient activation. Other important factors were: older age, being retired, poor health literacy, health-related quality of life, and social support. Total number of self-reported comorbidities and the perceived impact of comorbidities were also important for patients with more than one long-term condition.

Patient activation scores were reasonably enduring over time (*r* = 0.43 between baseline and at six months), although nearly half changed ‘levels’ of activation over that time. Few variables predicted change in activation over 6 months.

**Conclusions:**

This is the first large scale assessment of patient activation in the UK. Our data may be useful in identifying patients who need support with patient activation, and allow interventions (such as health coaching) to be tailored to better support older patients with long-term conditions who have symptoms of depression, poor social support and impaired health literacy. Further analyses of longitudinal studies will be necessary to better understand the causal relationships between patient activation and variables such as depression.

## Background

Managing long-term conditions and multimorbidity are key challenges for health care systems, and require a ‘whole systems perspective’ involving patients, practitioners, and service reorganisation [1]. The Chronic Care Model is an well-known exemplar of such a perspective [2].

Supported patient self-management is a widely used strategy for trying to address the challenges of managing long-term conditions and multimorbidity, including helping patients to change lifestyle behaviours and to learn core skills, such as responding to symptoms, managing medicine, improving diet, increasing exercise, stopping smoking, and managing interactions with professionals [3]. In the Chronic Care Model, people who achieve this are described as a ‘informed, activated patients’ [4].

The challenge of supporting self-management has been tackled in a variety of ways. Although information and education are necessary, they are rarely sufficient, and greater focus has been placed on behaviour change. However, even this may not be sufficient [5] and there has been increasing interest in the role of psychological variables relating to the attitudes and perceptions of the patient. *Self-efficacy* (a sense of control over actions and outcomes) has been hypothesised to be a key driver, [6] and was the basis for the Chronic Disease Self-Management Programme in the US (styled the Expert Patients Programme in the UK) [7]. Self-efficacy was initially hypothesised to be a potential *mediator* of the benefits of interventions for long-term conditions: [8, 9] that is, changes in self-efficacy were the mechanism by which the benefits were actually achieved [5]. Evidence also suggests that self-efficacy can be a *moderator* of outcomes: that is, it predicts which patients may benefit. Low baseline self-efficacy predicted additional benefit from the Expert Patients Programme [10].

Given these important effects, there is ongoing interest in the identification of patient characteristics that can help understand which patients might benefit most from supported self-management. This would allow services to better understand patient needs, target support, and measure quality. One such characteristic is *patient activation*.

### Patient activation

Patient activation is defined as how well a patient understands their own role in the healthcare process; and their level of knowledge, skill, and confidence in managing their own health [11]. Hibbard et al. (2005) describes four stages or levels of patient activation which range from those who are passive recipients of care, unable to self-manage, to those who are effective self-managers but may need some additional support during times of stress or crisis [12].

Improving patient activation is increasingly seen as an important component of new strategies to reform healthcare and improve health outcomes [13]. There is a growing body of evidence that patients who are more ‘activated’ have better health outcomes as well as an improved care experience [8]. In a US sample of mostly female patients with moderate to severe depression, level of patient activation was found to predict remission or improvement from depression over 12 months [14]. Depressed patients who were better activated were also more likely to engage in healthy behaviour changes such as quitting smoking and losing weight, and more likely to attend smear tests and mammogram screening [14].

NHS England (the body of the UK Department of Health which plans and oversees delivery of the English NHS) has identified patient activation as a potentially useful tool in the development of services to meet the needs of patients with long-term conditions [15]. Despite the interest in patient activation, there is relatively little data from UK patients. We use an existing cohort of patients aged 65+ with long-term conditions to describe levels of patient activation in the cohort, and to explore factors that predict change in activation over time.

## Method

We conducted a prospective cohort study of older people as part of the Comprehensive Longitudinal Assessment of Salford Integrated Care (CLASSIC) study. Eligible participants were those aged 65 years or older who were registered as having at least one long-term condition at a general practice in Salford (a city in the North West of England). Salford has a population of 294,916 (34,000 aged over 65 years) and a total of 52 general practices which are clustered in 8 neighbourhoods. Forty seven general practices in Salford were invited to take part in the study and 33 (65 %) agreed to participate.

We used the FARSITE software (a tool for recruitment to research studies in primary care - http://nweh.co.uk/products/farsite) to generate a list of eligible patients for each participating general practice. Each practice was then asked to identify any patients who met the exclusion criteria (patients in palliative care, and those with conditions which reduce capacity to consent and participate). Practices did not receive any incentive to take part but did receive support costs to reimburse them for their time.

A total of 12,989 patients were identified as eligible and were therefore mailed a questionnaire between November 2014 and February 2015. If they did not return the first questionnaire they were sent a reminder letter and a second copy of the questionnaire 3 weeks later. Participants were offered a £10 voucher on completion of the questionnaire as an incentive. Follow-up questionnaires were sent 6 months later (there was no incentive for completion of follow-up) to allow exploration of change over time.

### Outcome measure - patient activation

We used the 13 item version of the Patient Activation Measure (PAM) [13]. The PAM consists of 13 statements relating to patients beliefs about health care, confidence in their management of health related tasks, and self-assessed knowledge of their condition [13]. For each statement patients are required to say how much they either agree or disagree on a response scale of 1–5, where 1 represents “strongly disagree”, 4 represents “strongly agree” and 5 indicates that the statement is “not applicable” to them. A standardised spreadsheet in excel is used to score the PAM. Responses are used to generate a continuous score from 0 to 100 where higher scores indicate that the patient is more activated [13]. Where participants have answered that a statement is not applicable to them the data is treated as missing. A total score is generated where participants have answered at least 10 out of the 13 questions.

The continuous PAM scores are then categorised into four levels for descriptive purposes using the standardised excel spreadsheet. Those who fall into Level 1 are defined as passive recipients of care who do not understand that they can play an active role in their own healthcare. Level 2 includes patients who lack the basic knowledge and confidence to effectively self-manage (for example they may not understand the treatment options available to them or what their medications do). Level 3 includes those who have a basic knowledge about their health but they lack the confidence and skills to engage in positive self-management behaviours. Level 4 is for patients who have the knowledge and confidence to self-manage but who may need support during times of personal stress or health crisis [12].

The PAM has been found to be a valid and reliable measure in people with long-term conditions, such as those attending cardiac rehabilitation,[11] and in those patients with multimorbidity [16]. We used the continuous PAM score in the analyses.

### Predictor variables

- Socio-demographics
  We assessed age, gender, current work status and qualifications using questions taken from the General Practice Patient Survey [17]. We also asked participants to tell us if they lived alone, or with a spouse/partner, children (over or under 18 years old). Ethnicity was assessed using the 17 categories from the 2011 Census.
  Health literacy was assessed using the Single Item Literacy Screener (SILS) which asks patients to report how often they need help with reading information, pamphlets, or other written material from their doctor or pharmacy [18]. This question has demonstrated good reliability and validity and been previously used in adults with long term conditions [18, 19].
- Long-term conditions and multimorbidity
  We used a questionnaire to measure number self-reported long-term conditions for each patient and their impact on daily life [20]. Participants were given a list of 24 common long-term conditions and asked whether or not they had each condition and if they did how it interfered with their daily life (5 point scale ranging from 1 ‘not at all’ to 5 ‘a lot’). The conditions were: asthma; cancer; back pain; COPD; CKD; IBS; heart failure; depression and anxiety; diabetes; hearing problems; vision problems; heart disease; high blood pressure; high cholesterol; osteoarthritis; rheumatoid arthritis; osteoporosis; obesity; poor circulation; rheumatic disease; stomach problems; stroke; thyroid disorder; and other.
- Health related quality of life
  We used the EuroQol EQ-5D-5 L health utility index as a measure of health related quality of life [21]. The EQ-5D is a measure of general HRQoL which consists of the EQ-5D descriptive system and the EQ Visual Analogue Scale (EQ VAS). The EQ-5D has 5 domains: mobility, self-care, usual activities, pain/discomfort, and anxiety/depression. Participants are asked to record their health today for each domain where 1 indicates that they have no problems in that domain and 5 indicates that they have extreme problems. We used the recommended crosswalk tool to map the EQ-5D-5 L responses to validated utility scores based on the previous 3-level version of the EQ-5D [22]. The EQ-5D-5 L has been found to be a valid and reliable extension to the 3 level system [23] and has better measurement properties than the 3 level system for patients with long-term conditions and multimorbidity [24].
- Depression
  The co-existence of depression alongside other long-term conditions is prevalent and associated with significant impacts on health and costs [25]. The Mental Health Inventory-5 (MHI-5) was used to assess patient’s mood [26, 27]. The MHI-5 is a 5 item measure which asks patients to rate how much of the time they have felt happy, calm, nervous, or downhearted over the previous month. The measure has been well validated for identifying symptoms of depression [28, 29]. We used a cut off of a score of 60 to indicate presence of symptoms of depression, where a higher score indicates better mental health [29].
- Social support
  The ENRICHD Social Support instrument (ESSI) is a 7 item scale which was used to measure social support [30]. The scale covers partners, tangible help and emotional support. A total score is calculated by summing all individual items; a higher score indicates greater social support.
- Perceived impact of multimorbidity
  Where patients have more than one long-term condition, they often face significant logistical and emotional challenges in managing the burden of their *treatment*, as well as the burden of disease [31, 32]. For participants who self-reported two or more long term conditions we used a 16 item version of the MULTIPleS scale of illness perceptions [33]. MULTIPleS is a 22 item scale designed to measure the perceived impact of multimorbidity on patients emotional representations, and perception of treatment burden, prioritising conditions, causal links and activity limitations. However, for the purpose of this study we have removed the emotional representation subscale as this would confound with other psychological variables and reduced the scale to 16 items. Participants were asked to state how much they agree these 16 statements on a four point scale where 0 indicates strong disagreement and 3 indicates strong agreement. A summary score was then calculated where a higher score indicates greater perceived impact of multimorbidity. There is evidence that illness perceptions about multimorbidity predict outcomes [34].

### Statistical analysis

Analysis was restricted to patients for whom a valid PAM score could be derived. In the baseline questionnaire there was an error in the presentation of the PAM questionnaire in that the ‘*not applicable’* option was omitted from eight out of the 13 items. However, ‘*Not applicable’* was only chosen by 5 % of respondents where it was available as an option and therefore the impact of this is likely to be small.

There was missing data for some patients across some independent variables. To address this, for cases where no more than one independent variable across the full set was missing, logistic regression (linear, binary logistic, ordinal logistic or multinomial as appropriate), was used to impute the missing information, using the other eight variables, plus the baseline PAM score, as predictors. In so doing, 513 extra patients were included in the analyses. Missing data on the PAM questionnaire was not imputed.

Descriptive statistics are used to characterise the distribution of PAM scores and PAM levels, and changes in activation scores over time. Multiple linear regression was used for all analyses. Initially, we investigated associations between baseline PAM scores and independent variables. We examined Variance Inflation Factors (VIF) across all models to eliminate collinear variables. All VIF were below 10 and no variables were dropped from the analyses on the basis of VIF. Both raw and standardised regression coefficients are presented to determine the relative importance of each independent variable in addition to the absolute effect. Prospective multiple regression analyses considered whether these independent variables were predictive of change in PAM scores over the 6 months between the baseline and follow-up questionnaires. We used analysis of change scores as this approach is less bias than analysis of outcome at 6 months for use with observational data [35]. All regression analyses were clustered by general practice.

We repeated the analyses outlined above for the subset of patients with at least two long-term conditions (multimorbidity). The MULTIPleS scale was included as an additional independent variable in these analyses. Mean item imputation was used in order to calculate a MULTIPleS score for those patients with two or fewer items missing out of the original sixteen MULTIPleS items.

## Results

### Descriptive data on PAM scores

**Fig. 1:**
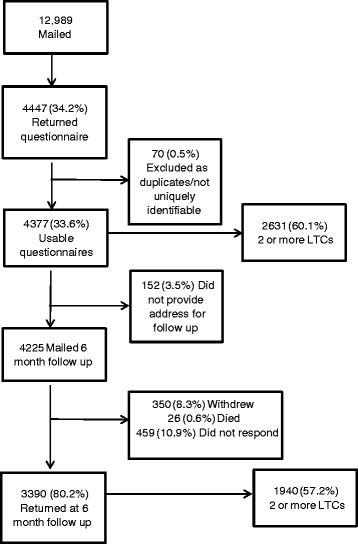
Flow of participants

The flow of participants is shown in Fig. 1. At baseline 4377 out of 12,989 (33.6 %) people returned a questionnaire. At 6 month follow up, 4225 were eligible to be mailed and 3390 (80.2 %) were returned.

Participant characteristics at baseline are shown in Table 1. The majority of participants were female, White British, and retired or not otherwise working. Symptoms of depression and anxiety were reported by 42.6 % of participants and 60.1 % reported having 2 or more long-term conditions.

**Table 1:** Participant characteristics

Continuous Variables	*n*	Mean	SD
Age	4098	75	6.8
*PAM Score*			
Baseline	3539	60.83	15.4
6 month Follow Up	2795	60.33	20.0
Depression	4123	67.1	22.4
*MULTIPleS* ^*a*^			
Summary Score	1703	35.6	9.1
Treatment Burden	1836	11.3	3.5
Prioritisation	1898	11.1	2.9
Causal Role	1831	6.3	2.2
Activity Restriction	1928	7.0	2.5
	*n*	%	–
*Gender*			
Male	2023	46.6	
Female	2315	53.4	
*Number of LTC*			
1 LTC	1115	25.5	–
2 or more	2631	60.1	
*Ethnicity*			–
White British	4123	95.4	
*Living alone*			–
Yes	1594	36.9	
No	2731	63.1	
*Employment status*			
Working	206	4.9	–
Retired/not economically active	3985	93.8	
Other	57	1.3	
*Health Literacy* ^*b*^			–
Never needs help	2974	70.3	
Rarely needs help	427	10.1	
Sometimes needs help	455	10.8	
Often needs help	158	3.7	
Always needs help	215	5.1	
*Social Support*			–
Poor social support	1392	33.6	
Good social support	2755	66.4	

^a^MULTIPleS only for those who self-reported 2 or more LTCs

^b^Single Item Literacy Screener (SILS)

A PAM score could be computed for a total of 3636 (83.1 %) participants at baseline. The mean PAM score was 60.7 (sd 15.4) with the majority of participants reporting PAM Level 3 (*n* = 1685, 46.3 %). Table 2 shows participant characteristics by PAM level.

**Table 2:** Participant characteristics by level of patient activation

Demographic Variable	PAM Level									
	Level 1		Level 2		Level 3		Level 4		Total *n*	
	*N*	%	*N*	%	*N*	%	*N*	%	*N*	%
*Gender*										
Male	253	14.7	288	16.7	821	47.6	362	21.0	1724	47.8
Female	332	17.6	353	18.7	857	45.4	344	18.2	1886	52.2
*Living alone*										
Yes	234	18.1	229	17.7	609	47.1	221	17.1	1293	35.9
No	351	15.2	411	17.8	1070	46.3	480	20.8	2312	64.1
*Employment status*										
Working	13	7.6	24	13.9	86	50	49	28.5	172	4.6
Retired or not economically active	553	16.5	603	17.9	1552	46.3	646	19.3	3554	94.3
Other	15	36.6	4	9.8	17	41.5	5	12.2	41	1.1
*Health Literacy*										
Never or rarely need help	317	11.2	494	17.4	1392	48.9	639	22.5	2842	80.5
Sometimes or often need help	108	29.1	77	20.8	150	40.4	36	9.7	371	10.5
Always need help	144	45.3	59	18.6	90	28.3	25	7.9	318	9.0
*Social Support*										
Poor social support	251	22.1	226	19.9	517	45.5	142	12.5	1136	32.7
Good Social support	318	13.6	397	16.9	1092	46.6	535	22.8	2342	67.3
*Depression*										
Not Depressed	180	8.8	309	15.1	1051	51.4	504	24.6	2044	57.2
Depressed	393	25.7	333	22.8	605	39.6	198	12.9	1529	42.8
*Number of LTC*1										
LTC 2 or more	103	11.2	149	16.23	451	49.2	214	23.3	917	28.6
	446	19.5	443	19.4	1019	44.6	376	16.5	2284	71.4
Continuous Variables	Mean	SD	Mean	SD	Mean	SD	Mean	SD	Mean	SD
Age in years	76.4	8.1	74.9	6.7	74.3	6.5	73.1	6.1	75	6.8

### Change in PAM scores over 6 months

The overall correlation between baseline and follow up PAM score was *r* = 0.43, *p* = 0.00. For those participants who had a complete PAM score at baseline total PAM scores varied between baseline and follow-up, decreasing or increasing by up to 50 points (Table 3). For over half of participants (52 %, *n* = 1392), PAM level remained stable between the baseline and 6 month follow-up questionnaires, increasing for 27 % (*n* = 718) of participants and decreasing for 21 % (*n* = 571). Participants who scored at PAM level 4 on the baseline were those more likely to remain at level 4 than to decrease by 6 month follow-up. Those who scored PAM levels 2 and 3 at baseline were the groups most likely to increase their scores at follow-up with 40 % (*n* = 188) at level 2 reaching level 3 at follow up and another 10 % moving up 2 levels to level 4 (Table 3).

**Table 3:** Change in patient activation level over 6 months

PAM Level at Baseline	PAM Level at 6 Month Follow Up				
	1	2	3	4	Total
1	204 52.4 %	78 20.1 %	91 23.4 %	16 4.1 %	389 100 %
2	93 19.8 %	142 30.2 %	188 40 %	47 10 %	470 100 %
3	85 6.7 %	176 13.9 %	707 55.9 %	298 23.5 %	1266 100 %
4	8 1.4 %	23 4.1 %	186 33.5 %	339 61 %	556 100 %
Total	390 14.6 %	419 15.6 %	1172 43.7 %	700 26.1 %	2681 100 %

### Predictors of Patient Activation at baseline

At baseline, patient activation was significantly lower in older patients, those with depression, and those with poor health literacy. Patient activation was higher in those with good quality of life, living alone and with better social support (Table 4). Depression had the strongest association with patient activation. Any self-reported impairment with health literacy was associated with lower activation scores; there was little difference in magnitude of effect between rarely needing help when reading medical literature and always needing help.

**Table 4:** Results from multivariate regression analysis of patient activation score at baseline

	All Participants ( *N* = 3293)				Participants with >=2 LTCs (*N* = 1563)			
Independent variable	b	95% CI	Beta (β)	*p*	b	95% CI	Beta (β)	*p*
Age	-0.19	-0.26 to -0.11	-0.08	0.000	-0.16	-0.25 to -0.07	-0.08	0.001
*Gender*								
Male								
Female	-0.72	-1.47 to 0.47	-0.02	0.095	-0.40	-1.65 to 0.86	-0.01	0.523
*Living status*								
Living with others								
Living alone	1.72	0.67 to 2.89	0.05	0.015	1.14	-0.63 to 2.91	0.04	0.199
*Work status*								
Employed								
Retired	-0.31	-1.94 to 1.32	0.00		2.36	-2.25 to 6.97	0.03	
Other	2.45	-3.36 to 8.26	0.00	0.437	6.67	-3.15 to 16.49	0.05	0.327
*Health literacy*								
Never needs help								
Rarely needs help	-4.54	-6.09 to -2.98	-0.09					
Sometimes needs help	-4.58	-6.25 to -2.90	-0.09		-3.57	-5.66 to -1.48	-0.08	
Often needs help	-5.12	-7.82 to -2.41	-0.06		-5.61	-8.61 to -2.61	-0.08	
Always needs help	-9.56	-12.80 to -6.32	-0.13	0.000	-9.86	-13.94 to -5.78	-0.17	0.000
Number of LTC	0.03	-0.27 to 0.32	0.00	0.846	0.72	0.31 to 1.13	0.08	0.001
*Depression*	0.09	0.06 to 0.12	0.13	0.000	0.08	0.04 to 0.12	0.11	0.001
HRQoL	9.56	6.71 to 12.40	0.16	0.000	4.95	0.93 to 8097	0.09	0.017
Social Support	4.32	3.14 to 5.50	0.13	0.000	3.73	1.78 to 5.68	0.12	0.000
MULTIPleS	-	-	-	-	-0.27	-0.40 to -0.13	-0.16	0.000
	R^2^ 0.168, Adjusted R^2^0.164				R^2^ 0.176, Adjusted R^2^ 0.169			

### Predictors of patient activation at baseline in patients with multimorbidity

In the subset of participants who self-reported 2 or more long-term conditions, depression, being older, impaired health literacy, HRQoL, social support, number of self-reported comorbidities, and a greater perceived impact of multimorbidity, were all significantly associated with patient activation scores at baseline. Living alone and being retired or not working were not significant for those with multimorbidity (Table 4).

### Predictors of change in patient activation scores over 6 months

Multivariate regression analysis of change in patient activation scores over 6 months across the whole sample did not identify any significant predictors of patient activation. In the subsample of patients with multimorbidity being depressed and being retired were significant predictors of change in patient activation scores (Table 5).

**Table 5:** Results from multivariate regression analysis of change in patient activation scores over 6 month follow up

	All Participants (*N* = 2709)				Participants with >=2 LTCs (*N* = 1273)			
Independent variable	b	95% CI	Beta (β)	*p*	b	95% CI	Beta (β)	*p*
Age	0.03	-0.11 to 0.16	0.01	0.678	0.01	-0.12 to 0.15	0.01	0.851
*Gender*								
Male								
Female	-0.23	-1.78 to 1.32	-0.01	0.762	-0.76	-2.85 to 1.33	-0.02	0.466
*Living status*								
Living with others								
Living alone	-0.40	-2.21 to 1.40	-0.01	0.654	1.04	-0.72 to 2.80	0.03	0.239
*Work status*								
Employed								
Retired	-3.44	-8.03 to 1.15	-0.04		-6.89	-12.74 to -1.03	-0.08	
Other	-4.59	-13.32 to 4.14	-0.02	0.259	-1.00	-12.88 to 10.88	-0.01	0.03
*Health literacy*								
Never needs help								
Rarely needs help	0.21	-2.66 to 3.10	0.00		-0.22	-3.43 to 2.99	0.00	
Sometimes needs help	0.53	-1.44 to 2.50	0.01		-0.07	-2.77 to 2.62	0.00	
Often needs help	-1.25	-5.40 to 2.90	-0.01		-2.05	-6.99 to 2.89	0.03	
Always needs help	-1.20	-4.61 to 2.25	-0.11	0.824	-2.29	-6.12 to 1.54	0.03	0.665
Number of LTC	0.29	-0.20 to 0.78	0.02	0.242	-0.08	-0.82 to 0.64	0.01	0.807
Depression	0.33	-0.01 to 0.08	0.04	0.137	0.05	0.01 to 0.09	0.07	0.009
HRQoL	-3.84	-8.15 to 0.46	-0.05	0.078	-0.46	-5.41 to 4.50	-0.01	0.852
Social Support	-3.20	-1.90 to 1.25	0.01	0.683	0.73	-1.83 to 3.30	0.02	0.566
MULTIPleS	-	-	-	-	0.08	-0.04 to 0.21	0.04	0.160
	R^2^ = 0.00, Adjusted R^2^ = 0.00				R^2^ 0.01, Adjusted R^2^ = 0.00			

## Discussion

### Statement of principal findings

Although pilot data is being collected on the use of the PAM, there is only limited published data on mixed samples of less than 400 patients [36]. Using a large cohort of older patients with long-term conditions, we explored demographic and health related factors associated with patient activation scores. We found that the most prominent factor associated with patient activation is depression in both the group of patients with one long-term condition and the subgroup of patients’ with multimorbidity. Other factors associated with patient activation were: older age, being retired, poor health literacy, health-related quality of life, and social support. The number of self-reported comorbidities and perceived impact of multimorbidity were also important for those with more than one long-term condition. However, the effects of all factors (except depression) were small implying that their impact on patient activation may not be clinically significant.

We explored predictors of change in patient activation scores over 6 months. We did not identify any significant predictors across the whole sample. However, in the subset of patients with multimorbidity both depression and being retired or not working significantly predicted change in patient activation scores.

### Strengths and weaknesses of the study

Study strengths included a large sample size, comprehensive measurement of patient-reported factors, and longitudinal measurement allowing assessment of predictors of change in patient activation scores.

The cohort achieved a 34 % response rate. This is similar to other studies in these populations using similar methods, [25, 34, 37] but leaves significant potential for non-response bias. The lack of data on non-respondents makes it difficult to assess direction and magnitude of bias. The data are therefore not a strong basis for assessments of prevalence, and data such as the proportions at each level of patient activation should be used with caution. Assessments of the relationships between measures may be less vulnerable to non-response bias. Completion of follow up was over 80 % and thus the potential for bias here is less, although some scales (such as MULTIPleS) did suffer additional missing data. The cohort is also limited to older patients and those living in one area in the UK, which means that the results may not generalise. Long-term conditions were self-reported and we did not confirm diagnoses with medical records.

### Comparison with other published data

Previous work in a cross sectional study found lower patient activation scores to be associated with symptoms of depression and poor quality of life [38]. Our study extends this by showing that depression predicts change in patient activation scores over 6 months. A longitudinal study found that patients with symptoms of depression, even when subclinical, were less likely to improve their activation scores or to engage in self-management behaviours over 6 months [39]. A large longitudinal study of patient activation in US patients with moderate to severe depression found that those with more severe depression tended to be less activated and that higher patient activation at baseline predicted greater reduction in depression over 1 year [14]. Those patients with the highest patient activation scores at baseline had over twice the odds of remission from depression.

In a cross sectional survey of US patients with multiple sclerosis, patients’ level of education, as well as depression, was found to be associated with patient activation [40]. Lower levels of education have previously been shown to be associated with poor health literacy [41] which we found to be significantly associated with patient activation in our sample of older patients. A large Danish cohort study of over 29,000 patients has shown that people with long-term conditions have greater difficulties in understanding health information than the general population and have greater problems choosing and engaging with health care [42, 43].

We found that the total number comorbid long-term conditions was associated with patient activation in the short term, but did not predict change in activation prospectively. A smaller US study of 850 patients with multimorbidity over the age of 65 also found that number of comorbid conditions was not related to patient activation [16]. Although a crude count of the *numbers* of conditions may not predict activation, we found that patient experience of multimorbidity, as measured by MULTIPleS, was associated with patient activation and had the second strongest association after depression.

Social support had a strong association with patient activation across all patients. In a large cross sectional survey of Danish patients with Type 2 diabetes greater contact with friends was associated with higher patient activation and having a poor functional social network was associated with lower patient activation [44].

We found that living alone was associated with higher patient activation in the short term. A systematic review of studies on chronic illness self-management in people living alone found that patients who live alone often ensure that they actively attend to their health needs as they are required to manage their health independently and often have no immediate help available to them [45]. Furthermore, those who live alone have been shown to value social support outside the home and may actively seek and maintain better social connections than those with greater support in the home [45].

We found patient activation scores at 6 months were moderately correlated with baseline scores, with fairly significant movement between levels. It is complex to interpret this change data, as it is not known what clinical or other health and social care interventions were received by patients. The entire local health economy was undergoing a major re-organisation for this age group, but linking that to changes in patient scores would be challenging. The data do suggest some caution in using patient activation scores for very specific targeting of interventions, given patient movement between levels of activation.

### Meaning of the study: possible mechanisms and implications for clinicians or policymakers

Our data suggest a cluster of characteristics that are associated with low activation, mainly depression but also other demographic, social, educational, and health factors. If effective interventions are developed in the UK to improve patient activation, these patients, and particularly those with depression, may be usefully prioritised.

Additionally, the changeable factors which are associated with patient activation may suggest the potential content of interventions. For example, interventions or programmes will need to provide assistance with health literacy (through education) and depression symptoms (through web-based support or cognitive-behavioural therapy interventions), and may usefully involve a component around social support, particularly around increasing functional social support. The associations with patient experience of multimorbidity (MULTIPleS) highlight the potential importance of the assessment of this issue in patients [46].

### Unanswered questions and future research

This study is unique because it presents the first assessment of depression and patient activation in older adults with long-term conditions. We found that depression was significantly associated with patient activation and, for those patients with multimorbidity, it predicted change in patient activation scores over 6 months. However, the opposite pattern of findings has been shown in the literature; patient activation predicted remission, response and changes in depression scores over 12 months in a large US sample of patients with moderate to severe depression [14]. Thus, an unresolved research challenge is to fully understand whether lower patient activation precedes depression or depression leads to lower levels of patient activation. The examination of the temporal links between depression and patient activation as part of a longitudinal study with multiple follow-up assessment points is a fruitful approach to disentangle the relationship between depression and patient activation. This knowledge has the potential improve the effectiveness of self-management interventions by timely and appropriate management of depression and patient activation.

Moreover, much of the research to date on patient activation has assessed the degree to which it is associated with key outcomes, such as quality of life and costs. Such might suggest two more specific causal functions of activation.

First, activation may be a *mediator* of other outcomes, [8] driving other outcomes. Intervening to improve activation may have effects on other outcomes further down the causal pathway. For example, a self-management intervention that raises a patient’s level of activation may help them to better manage their long-term condition in the short-term, leading to longer-term reductions in costs and quality of life. Methods to assess mediation are available, although they are complex, and often most usefully done in the context of a trial [47].

Secondly, activation may be a *moderator* of other outcomes [8]. If this is the case, then levels of activation will influence the benefit that patients report from other interventions, and assist in targeting. For example, a low-intensity web-based self-management intervention may demonstrate good outcomes with patients with a higher level of activation, but may be ineffective with those with lower activation, where a more intensive intervention with greater professional input may be required. Again, displaying such effects is possible but complex, and a rigorous demonstration may require subgroup analyses in a large trial or pooled analyses across several trials [48]. The high levels of change in patient activation may limit is utility as a moderator.

## Conclusions

Older patients in the UK show variation in their levels of activation. Activation is low in depressed, older, retired patients with poor health literacy, and those who lack social support. If activation is proven to be a useful concept for targeting of interventions, our data may be useful in identifying the sorts of patients who need activation, and may suggest hypotheses about ways of raising activation to improve outcomes. Further analyses of longitudinal studies will be necessary to better understand the causal relationships between patient activation and variables such as depression.

## Abbreviations

CLASSIC: Comprehensive Longitudinal Assessment of Salford Integrated Care
HRQoL: Health related quality of life
LTC: Long-term condition
MHI-5: Mental Health Inventory-5
NHS: National Health Service
PAM: Patient Activation Measure
SILS: Single item literacy screener
UK: United Kingdom
US: United States
